# Recurrent Stage 3 Sacral Pressure Injury in a Patient With Fistulizing Crohn's Disease: Clinical and Social Considerations

**DOI:** 10.7759/cureus.114973

**Published:** 2026-08-22

**Authors:** Jennifer Acevedo, Matthew Majer, Dayana Rojas

**Affiliations:** 1 Internal Medicine, St. George's University School of Medicine, St. George's, GRD; 2 Internal Medicine, American University of the Caribbean School of Medicine, Cupecoy, SXM; 3 Epidemiology and Public Health, Independent Researcher, Miami, USA

**Keywords:** continuity of patient care, crohn’s disease (cd), discharge planning, housing instability, inflammatory bowel disease (ibd), malnutrition, multidisciplinary care (mdc), pressure injury, social determinants of health (sdoh), ­wound healing

## Abstract

Pressure injuries are challenging chronic wounds because healing depends on systemic and patient-specific factors in addition to local wound care. Patients with Crohn's disease may be particularly vulnerable to impaired healing because chronic inflammation, nutritional compromise, prior intestinal surgery, infection, and limited mobility can interfere with tissue repair. Sustaining improvement after hospital discharge may be further complicated by social barriers that limit continuity of wound care.

We report the case of an adult man with advanced fistulizing Crohn’s disease who was admitted for evaluation of worsening drainage from a chronic vesicocutaneous fistula and was found to have a recurrent stage 3 sacral pressure injury. His medical history was significant for prior colectomy with colostomy creation, limited mobility, housing instability, and a sacral pressure injury that had previously healed before recurring. During hospitalization, the pressure injury was managed with regular wound assessment, dressing changes, frequent repositioning, oral nutritional supplementation, and multidisciplinary care, without evidence of worsening infection or a need for surgical intervention.

This case illustrates the importance of addressing biological risks during inpatient wound management while also considering whether evidence-based care will remain feasible after discharge. However, post-discharge outcomes were unavailable; therefore, the proposed benefit of incorporating housing and resource constraints into discharge planning warrants further study.

## Introduction

Pressure injuries are a substantial source of morbidity, particularly among medically complex patients. Healing depends on appropriate local wound management as well as systemic factors such as inflammation, nutritional status, mobility, and infection [1-4]. Although evidence-based inpatient care can promote improvement, maintaining that progress after discharge often depends on whether the patient can continue the treatment plan in the outpatient setting.

Crohn's disease creates additional challenges for wound healing through chronic inflammation, fistulizing disease, recurrent infection, prior intestinal surgery, and nutritional deficiencies [5-10]. Social risks may compound this vulnerability by making an otherwise appropriate outpatient treatment plan difficult to implement [3,11-15].

We present the inpatient course of a patient with advanced fistulizing Crohn’s disease and a recurrent stage 3 sacral pressure injury. This report focuses on wound management during hospitalization and the discharge-planning challenges associated with housing instability. The patient remained hospitalized at the time of writing, and post-discharge continuity of wound care and wound outcomes were therefore not assessed.

## Case presentation

A 62-year-old man with advanced fistulizing Crohn’s disease complicated by enterocutaneous and vesicocutaneous fistulas presented with several days of worsening foul-smelling drainage and surrounding erythema at a suprapubic fistula site. He denied fever, chills, nausea, or vomiting. His medical history included prior colectomy with colostomy creation, a previously healed stage 3 sacral pressure injury, chronic kidney disease, hypertension, chronic pain syndrome, limited mobility, and housing instability. His Crohn’s disease was being managed with mesalamine.

Day 1 was defined as the day of presentation and the beginning of a single continuous hospitalization. The patient was afebrile and hemodynamically stable on admission. Laboratory testing during hospitalization demonstrated mild leukocytosis and anemia. Computed tomography of the abdomen and pelvis showed a complex vesicocutaneous fistula extending from the bladder to the anterior abdominal wall without a drainable abscess. He was admitted for multidisciplinary management involving colorectal surgery, infectious disease, gastroenterology, urology, wound care, case management, and social work. Gastroenterology discontinued mesalamine after determining that it was unlikely to provide meaningful benefit in advanced fistulizing disease.

During the initial wound assessment, the patient was found to have a recurrent sacral pressure injury that was clinically classified as stage 3. The wound bed consisted predominantly of granulation tissue, with focal yellow slough and dark eschar at the margins that did not obscure the overall extent of tissue loss (Figure 1). No exposed bone or clinical evidence of osteomyelitis was present. The patient had experienced one previous sacral pressure injury, which had healed before this admission. Management of the recurrent wound included regular assessment, dressing changes, frequent repositioning, and consultation with the wound-care team.

**Figure 1:**
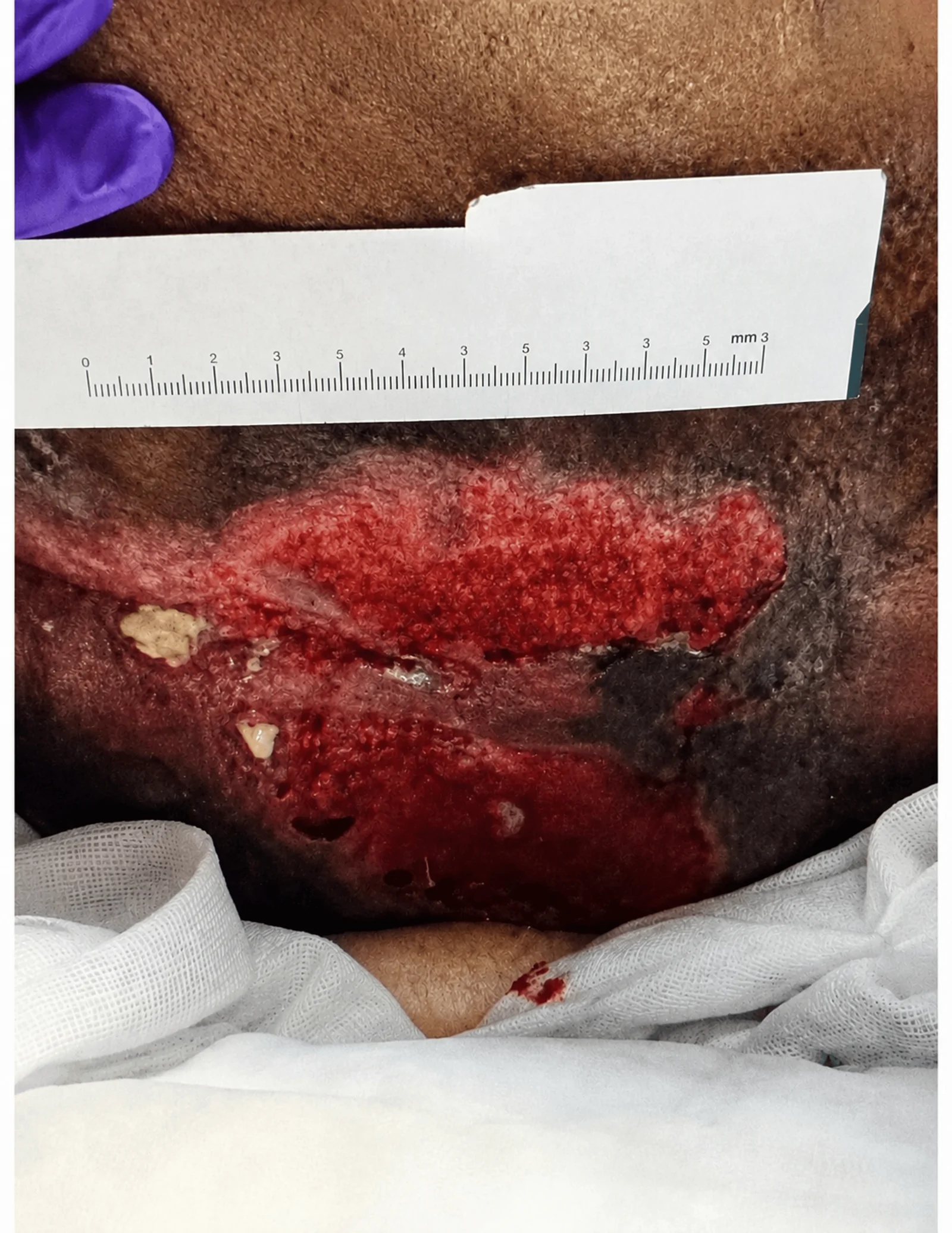
Clinical appearance of the recurrent stage 3 sacral pressure injury during hospitalization.

A lateral abdominal-wall cutaneous opening with an adjacent wound was also present, consistent with the patient’s known left vesicocutaneous fistula site (Figure 2). A deep culture obtained from the suprapubic fistula on Day 3 grew moderate methicillin-resistant *Staphylococcus aureus *(MRSA). Blood cultures obtained during the initial evaluation showed no growth after 100 hours, and imaging demonstrated no drainable abscess. In view of the patient’s clinical stability and chronic fistulous disease, Infectious Disease considered the wound-culture result more consistent with colonization than active infection and recommended monitoring without antibiotics.

**Figure 2:**
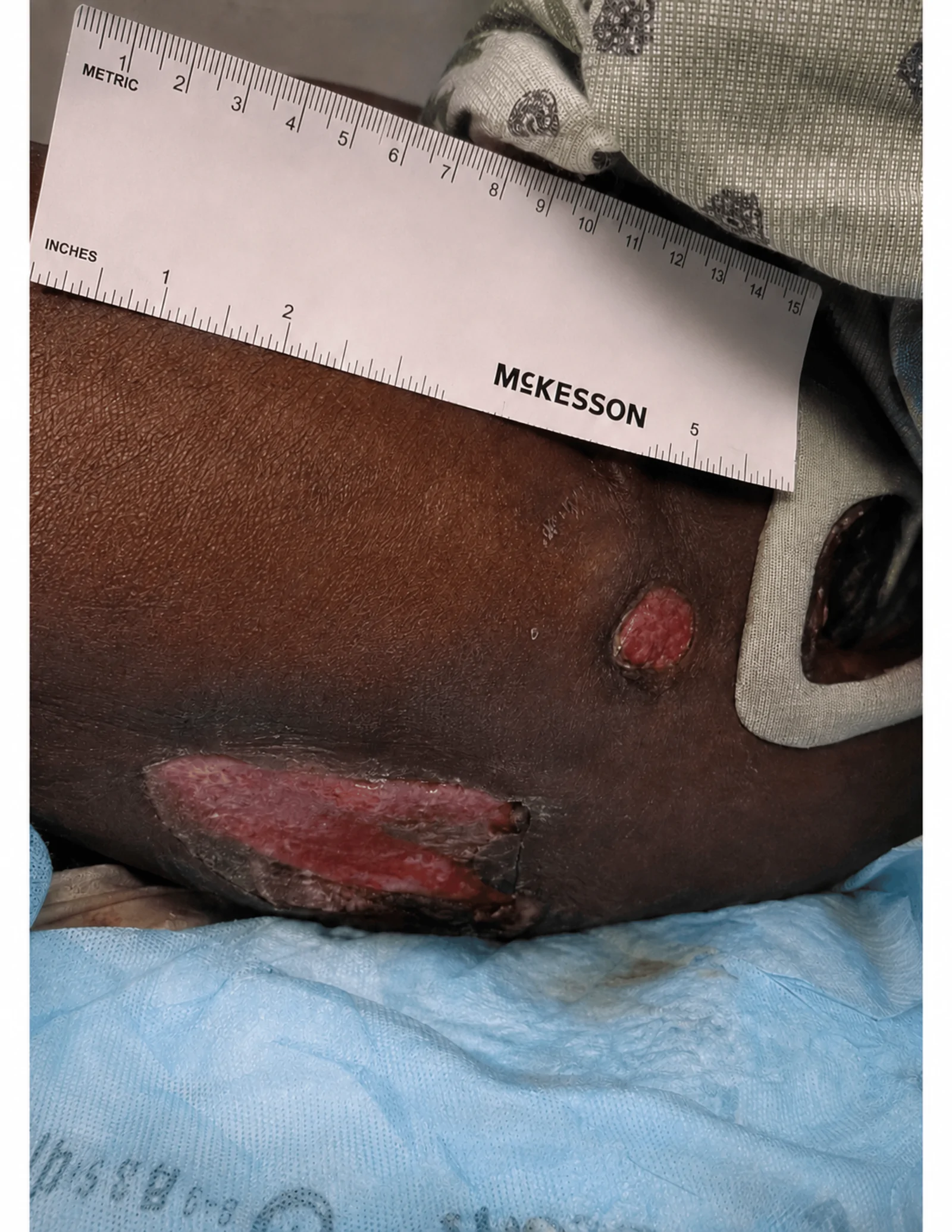
Clinical appearance of the left lateral abdominal-wall fistula site during hospitalization.

Nutritional supplementation was added on Day 24 and included Juven, one packet twice daily, and Nepro, one can with meals. The patient also received a regular-consistency diet restricted to 2 g of sodium and 2 g of potassium. Serum albumin increased from 2.5 g/dL on Day 43 to 3.0 g/dL on Day 47 and remained near that level through Day 50. Laboratory testing on Day 43 showed hyperchloremia, a serum carbon dioxide level of 12 mmol/L, and a normal anion gap, consistent with a non-anion-gap metabolic acidosis. Renal function remained stable, and the carbon dioxide level increased to 24 mmol/L by Day 49, supporting a transient metabolic disturbance rather than progressive renal dysfunction. Clinically relevant serial laboratory findings are summarized in Table 1.

**Table 1:** Clinically relevant serial laboratory findings from a single continuous hospitalization. Day 1 represents the day of presentation and admission. WBC, white blood cell count; BUN, blood urea nitrogen; CO₂, carbon dioxide

Hospital day	WBC (×10⁹/L)	Hemoglobin (g/dL)	Sodium (mmol/L)	Potassium (mmol/L)	Chloride (mmol/L)	CO₂ (mmol/L)	Anion gap (mmol/L)	BUN (mg/dL)	Creatinine (mg/dL)	Calcium (mg/dL)	Albumin (g/dL)
Reference range	4-11	13.5-17.5	136-145	3.5-5.1	98-107	21-31	5-15	7-25	0.7-1.3	8.6-10.3	3.5-5.7
Day 11	11.3	8.5	-	-	-	-	-	-	-	-	-
Day 26	10.7	10.1	-	-	-	-	-	-	-	-	-
Day 28	10.4	9.1	-	-	-	-	-	-	-	-	-
Day 34	11.7	8.6	-	-	-	-	-	-	-	-	-
Day 43	-	-	135	4.2	112	12	11	14	0.91	8.1	2.5
Day 44	-	-	136	3.9	108	17	11	13	0.87	8.2	2.5
Day 45	-	-	137	3.7	110	20	7	13	0.9	8.5	2.6
Day 47	-	-	138	3.9	105	19	14	15	0.91	8.8	3.0
Day 49	-	-	139	3.9	102	24	13	14	0.71	8.5	2.9
Day 50	-	-	139	3.8	106	24	9	13	0.7	8.6	3

Subsequent assessments of the sacral pressure injury showed no clinical evidence of worsening infection or need for surgical intervention. Discharge planning included coordination of outpatient follow-up with Gastroenterology for continued management of his Crohn’s disease, as well as with colorectal surgery and wound care. Case management and social work assisted with planning and placement. At the time of this report, the patient remained hospitalized while placement in a skilled nursing facility was being pursued because of his housing instability. Consequently, whether the proposed discharge plan would maintain continuity of wound care or preserve the patient’s inpatient wound status could not be evaluated.

## Discussion

The most informative aspect of this patient’s course was the management of a recurrent stage 3 sacral pressure injury alongside advanced fistulizing Crohn’s disease. The patient’s course highlights the importance of addressing modifiable components of wound care while also considering whether those measures can be continued beyond the inpatient setting.

Several features of this patient's disease likely contributed to impaired tissue repair. Advanced Crohn's disease and enteric fistulas can combine to cause persistent inflammation, recurrent infection, prior intestinal surgery, and nutritional compromise [5-10]. Nutritional status therefore required particular consideration. This patient had persistent hypoalbuminemia (2.5-3.0 g/dL), supporting concern for nutritional and inflammatory burden; however, serum albumin alone does not establish nutritional status. Patients with Crohn's disease remain at risk for malnutrition because of reduced oral intake, malabsorption, increased metabolic demands, and prior intestinal surgery [5-8]. Pressure injury guidance recommends comprehensive nutritional assessment rather than reliance on a single laboratory value [1-3,16].

Prior reports describe different relationships between gastrointestinal disease and sacral wounds. Wang et al. described a patient with Crohn’s disease in whom rectal perforation led to a presacral abscess, gluteal extension, and sacral osteomyelitis requiring drainage and fecal diversion [17]. Kubota et al. reported a refractory sacral pressure injury associated with small intestinal bacterial overgrowth and severe nutritional deficiency that improved after treatment of the underlying malabsorption [18]. In our patient, the enterocutaneous and vesicocutaneous fistulas were anatomically separate from the sacral pressure injury, and there was no drainable abscess or clinical evidence of osteomyelitis. MRSA isolated from the suprapubic fistula was considered colonization rather than active infection. This case is therefore notable for the management of a recurrent pressure injury alongside advanced fistulizing Crohn’s disease, with housing instability adding complexity to discharge planning.

Effective outpatient wound management requires dependable access to dressings, nutrition, pressure redistribution, transportation, and follow-up [3,12,13,15]. Social risks are common among patients with inflammatory bowel disease [11,12,14]. They have been associated with greater disease activity, medication nonadherence, unplanned healthcare use, financial toxicity, and reduced continuity [11-14]. Given these associations, housing instability represented a potential barrier to consistent wound care in this patient, but its contribution to the wound’s recurrence cannot be established.

The recurrence was likely multifactorial. Chronic inflammation, fistulous disease with ongoing infection risk, reduced mobility, nutritional compromise, and housing instability were all relevant considerations, although the relative contribution of each cannot be determined. For future patients with similar barriers, discharge planning could include referral to medical respite or temporary housing programs that provide a stable environment for post-acute recovery, as well as linkage to food assistance, transportation services, medication-access support, and community sources of dressing supplies [12,14,15,19]. Wound care in unhoused populations is complicated by environmental exposure, limited storage for supplies, difficulty maintaining hygiene, and inconsistent access to follow-up [15]. Medical respite programs are designed for patients who no longer require hospitalization but remain too medically vulnerable to recover safely in a shelter or on the street; available evidence suggests that these programs may reduce hospital admissions, inpatient days, and readmissions [19]. Although these services were not implemented in this case, early assessment of eligibility and availability may support continuity of wound care after discharge.

Discharge readiness requires more than clinical stability and a written wound-care plan. It also requires determining whether the patient can realistically carry out that plan. Early coordination among wound care, nutrition services, social work, case management, gastroenterology, and primary care may help identify and address barriers before discharge.

Post-discharge wound outcomes and long-term follow-up were unavailable, so this case cannot determine whether the inpatient wound status was maintained after discharge or how much housing instability contributed to recurrence. The discussion of post-discharge continuity is therefore based on clinical reasoning and existing literature rather than an outcome demonstrated in this patient.

## Conclusions

This case describes the inpatient management of a recurrent stage 3 sacral pressure injury in a patient with advanced fistulizing Crohn’s disease. It also highlights the practical concern that an appropriate wound-care plan may be difficult to continue after discharge without adequate resources and support. Post-discharge outcomes were unavailable, but clinical reasoning and existing literature support assessing whether patients have the resources needed to continue wound care after hospitalization.
